# Supplementary material for: High Tumor Mutation Burden Is Associated with Poor Clinical Outcome in EGFR-Mutated Lung Adenocarcinomas Treated with Targeted Therapy
Source: Biomedicines. 2022 Aug 29;10(9):2109. doi: 10.3390/biomedicines10092109 (PMC9495802; doi:10.3390/biomedicines10092109)
Supplement: Supplementary file 1 [file biomedicines-10-02109-s001.zip › biomedicines-1870316-supplementary.pdf]

Supplementary material

Materials and methods

1. DNA extraction and next generation sequencing (NGS) library preparation

For DNA extraction, four to ten formalin-fixed paraffin-embedded tissue sections of 10 µm thickness were cut and deparaffinized. DNA extraction from tissues was performed using the RecoverAll^{TM} Multi-Sample Isolation Kit (Thermo Fisher Scientific, Waltham, MA, USA) according to the manufacturer's instructions. The tumor mutation burden (TMB) was assessed using a 409-gene targeted NGS panel (Oncomine^{TM} Tumor mutation load Assay, Thermo Fisher Scientific). For NGS library preparation, 5–40 ng of DNA was used depending on the availability of the input material. The libraries were purified using Agencourt AmpureXP beads (Beckman Coulter, Brea, CA, USA) and quantified by qPCR using an Ion Universal Quantitation Kit (Thermo Fisher Scientific).

2. Sequencing and quality assessment

Sequencing runs were planned on the Torrent Suite Software^{TM} v5.10 and libraries were diluted to 50 pM, combined in batches of five libraries, loaded onto an Ion 540^{TM} chip using the Ion Chef^{TM} instrument, and sequenced on an Ion S5XL^{TM} platform (Thermo Fisher Scientific). Raw data were processed automatically on the Torrent Server^{TM} and aligned with the hg19 reference genome. Sequencing quality was determined according to thresholds of three parameters: mean depth ≥ 300X; number of reads ≥ 5,000,000, and uniformity ≥ 80%. Fifteen cases were excluded from the study because the sequencing quality of their samples did not meet the minimum requirements. The remaining 88 cases obtained an average of 10,172,722 (5,132,431–18,512,640) reads per sample, with 98.6% (94.1−99.4%) on-target rates, 95.6% (82.1−97.9%) read uniformity, and 670X (304X−1217X) average coverage. Sequencing data

were subsequently uploaded in the BAM format to the Ion Reporter^{TM} Analysis Server for TMB calculation and variant calling.

3. Handling for potential deamination artifacts

Variant detection and TMB calculations were performed using Ion Reporter^{TM} Analysis Software v5.10 (IR) using the Oncomine^{TM} Tumor Mutation Load w2.0 workflow. The deamination score, which reflects potential deamination artifacts, was automatically calculated using this workflow. Eighteen cases with a deamination score of more than 40 were considered to have severe deamination artifacts, and hence, were not reliably analyzed and excluded. The median deamination score of the remaining 88 patients was 2 (range, 0–37). The default limit of detection (LOD) was set at 5% allelic frequency (AF) and adjusted to 10%, depending on the deamination score (5% for the low deamination group [deamination score ≤2], and 10% for the high deamination group [deamination score >2]). Finally, we analyzed 88 cases (32 and 30 in the low and high deamination groups, respectively) for TMB calculation and variant detection.

4. TMB calculation

TMB calculation was performed using the Ion Reporter^{TM} Analysis Software v5.10 (IR) using the Oncomine^{TM} Tumor Mutation Load w2.0 workflow. Germline variants were filtered automatically by cross-referencing with the UCSC common SNPs (not in), ExAC (0.0 ≤ ExAC GAF ≤ 10^{-6}), 10,000 genomes (0.0 ≤ and ≤ 10^{-6}), and 5000 exomes (0.0 ≤ 5000 Exomes Global MAF [20161108] ≤ 10^{-6}) databases. Somatic variants with homopolymer stretches longer than 4 bp were also excluded. The default LOD was set at 5% allele frequency and adjusted to 10% depending on the deamination score. TMB was calculated by dividing the number of nonsynonymous (missense and nonsense) somatic single nucleotide variants (SNVs) and

coding indels by the number of exonic bases with at least 60x coverage and was expressed as the number of mutations per megabase. TMB values were rounded to two decimal places.

5. Variant detection

Variant detection was performed using Ion Reporter^{TM} Analysis Software v5.10 (IR). Germline variants were filtered automatically by cross-referencing with UCSC common SNPs (not in), ExAC (0.0 ≤ ExAC GAF ≤ 0.001), 10,000 genomes (0.0 ≤ and ≤ 0.001), and 5000 exomes (0.0 ≤ 5000 exomes global MAF [20161108] ≤ 0.001) databases. The required coverage was ≥ 200. The default LOD was set at 5% AF and adjusted to 10% depending on the deamination score. Variants, including missense or nonsense SNVs and indels in exonic locations, were listed as detected mutations.

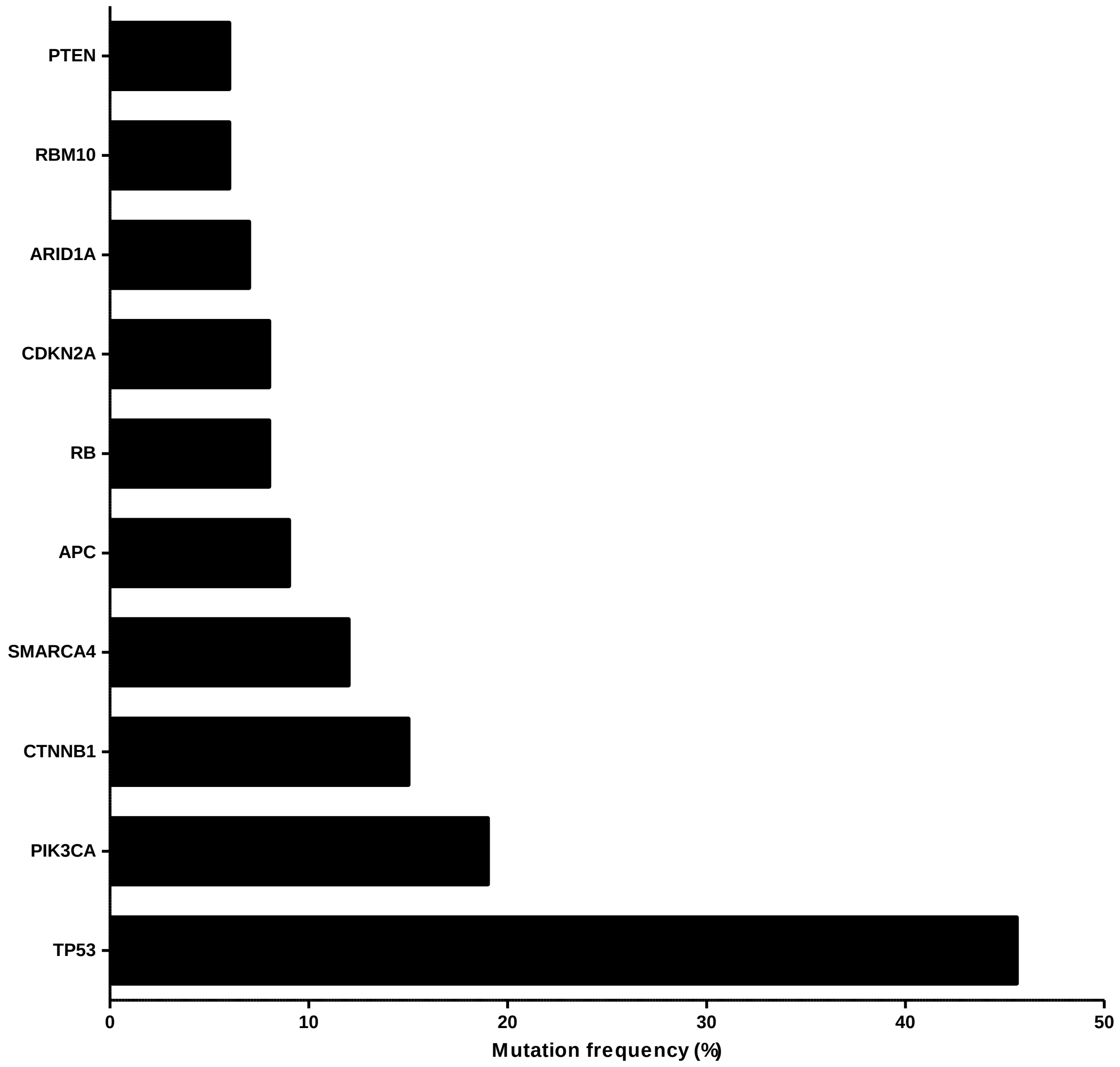

Supplementary Figure S1. The list of top 10 somatic mutations detected and their frequencies

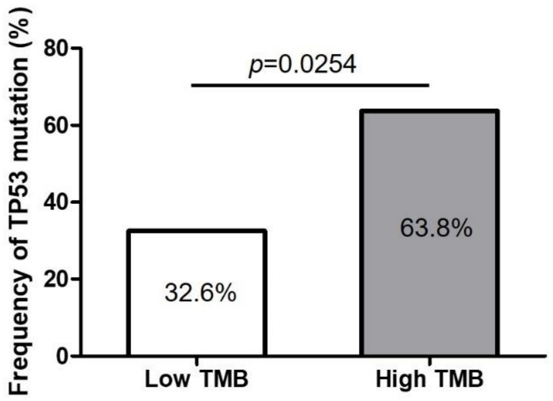

Supplementary Figure S2. Frequency of *TP53* mutation according to different TMB levels

Supplementary Table S1. Median TMB levels according to different clinicopathological parameters

	No. of patients (%)	TMB level*	*p*-value
All	88 (100.0)	3.4 (1.7-5.9)	
Age (years)			0.9334
<70	41 (46.6)	3.4 (2.5-5.1)	
≥70	47 (53.4)	3.4 (1.7-5.9)	
Sex			0.2518
Male	44 (50.0)	3.4 (2.1-5.9)	
Female	44 (50.0)	3.4 (1.7-5.1)	
Smoking			0.3099
Never	62 (70.5)	3.4 (1.7-5.9)	
Ever	26 (29.5)	4.2 (1.7-6.7)	
Smoking intensity			0.6232
<30 pack-years	72 (81.8)	3.4 (1.7-5.9)	
≥30 pack-years	16 (18.2)	3.8 (1.7-5.0)	
ECOG PS			0.8606
0, 1	70 (79.5)	3.4 (1.7-5.9)	
≥2	18 (20.5)	2.9 (1.7-5.0)	
Stage			0.0543
III	15 (17.0)	3.0 (1.4-3.9)	
IV	73 (83.0)	4.2 (3.4-6.8)	
Involved organs			0.1359
<3	67 (76.1)	2.5 (1.7-4.2)	
≥3	21 (23.9)	3.4 (2.1-5.1)	
Brain metastasis			0.4942
No	60 (68.2)	3.4 (1.7-5.9)	
Yes	28 (31.8)	3.4 (2.1-5.1)	
Liver metastasis			0.0030
No	74 (84.1)	1.7 (0.8-2.5)	
Yes	14 (15.9)	3.8 (2.5-5.9)	
EGFR subtypes			0.1797†

19del	52 (59.1)	3.4 (1.7-5.9)	
L858R	31 (35.2)	3.4 (1.7-5.0)	
Others	5 (5.7)	4.9 (4.0-5.9)	
First-line TKI			0.2537†
Gefitinib	14 (15.9)	3.4 (1.5-5.7)	
Erlotinib	6 (6.8)	3.4 (1.7-5.9)	
Afatinib	68 (77.2)	4.7 (4.0-5.5)	

*presented as median (interquartile range).
†Kruskal–Wallis test
ECOG PS, Eastern Cooperative Oncology Group Performance Status; EGFR, epidermal growth factor receptor; 19del, deletion mutation at exon 19; TKI, tyrosine kinase inhibitor; TMB, total mutation burden.

Supplementary Table S2. Salvage treatments after frontline EGFR-TKI failure (n=52)

	Low TMB (n=33)	High TMB (n=19)
T790M mutation		
Positive	17	5
Negative	16	14
Second-line treatment (n=52)		
Osimertinib	17	5
Pemetrexed	3	2
Pemetrexed/platinum	12	11
Etoposide/platinum	1	1
Third-line treatment (n=29)		
Pemetrexed/platinum	8	1
Paclitaxel	2	4
Paclitaxel/platinum	3	2
Pembrolizumab	3	2
Atezolizumab	1	1
Gefitinib	1	0
Erlotinib	1	0
